# Measurement of distal intramural spread and the optimal distal resection by naked eyes after neoadjuvant radiation for rectal cancers

**DOI:** 10.1186/s12957-022-02756-2

**Published:** 2022-09-14

**Authors:** Ge Sun, Xiaolong Ye, Kuo Zheng, Hang Zhang, Paul Broens, Monika Trzpis, Zheng Lou, Xianhua Gao, Lianjie Liu, Liqiang Hao, Edgar Furnee, Chenguang Bai, Wei Zhang

**Affiliations:** 1 Department of Colorectal Surgery, Changhai Hospital, Shanghai, China; 2 Department of Surgery, University Medical Center Groningen, University of Groningen, Groningen, the Netherlands; 3 Department of Pathology, Changhai Hospital, Shanghai, China; 4 Institute of Pathology, Southwest Hospital, Chongqing, China; 5 Key Laboratory of Tumor Immunopathology, Ministry of Education of China, Chongqing, China

**Keywords:** Intramural spread distance, Preoperative radiation therapy, Rectal cancer, Tumor margin, Oncological safety

## Abstract

**Background:**

The safe distance between the intraoperative resection line and the visible margin of the distal rectal tumor after preoperative radiotherapy is unclear. We aimed to investigate the furthest tumor intramural spread distance in fresh tissue to determine a safe distal intraoperative resection margin length.

**Methods:**

Twenty rectal cancer specimens were collected after preoperative radiotherapy. Tumor intramural spread distances were defined as the distance between the tumor’s visible and microscopic margins. Visible tumor margins in fresh specimens were identified during the operation and were labeled with 5 - 0 sutures under the naked eye at the distal 5, 6, and 7 o’clock directions of visible margins immediately after removal of the tumor. After fixation with formalin, the sutures were injected with nanocarbon particles. Longitudinal tissues were collected along three labels and stained with hematoxylin and eosin. The spread distance after formalin fixation was measured between the furthest intramural spread of tumor cells and the nanocarbon under a microscope. A positive intramural spread distance indicated that the furthest tumor cell was distal to the nanocarbon, and a negative value indicated that the tumor cell was proximal to the nanocarbon. The tumor intramural spread distance in fresh tissue during the operation was 1.75 times the tumor intramural spread distance after formalin fixation according to the literature.

**Results:**

At the distal 5, 6, and 7 o’clock direction, seven (35%), five (25%), and six (30%) patients, respectively, had distal tumor cell intramural spread distance > 0 mm. The mean and 95% confidence interval of tumor cell intramural spread distance in fresh tissue during operation was − 0.3 (95%CI − 4.0 ~ 3.4) mm, − 0.9 (95%CI − 3.4 ~ 1.7) mm, and − 0.4 (95%CI − 3.5 ~ 2.8) mm, respectively. The maximal intraoperative intramural spread distances in fresh tissue were 8.8, 7, and 7 mm, respectively.

**Conclusions:**

The intraoperative distance between the distal resection line and the visible margin of the rectal tumor after radiotherapy should not be less than 1 cm to ensure oncological safety.

## Background

Intraoperative determination of the safe distal resection line after radiotherapy in rectal cancer surgery remains a matter of controversy. The American Society of Colon and Rectal Surgeons (ASCRS) manual from 2019 proposes that the whole tissue identified as a tumor before radiotherapy should be removed based on pre-radiation tumor margins [1]. However, the question of how to optimally determine the pre-radiation tumor margin has not been answered by this manual. Shrinkage of the tumor and normal tissues will more or less pull up the distal tumor margin, and that is why using the length between the tumor margin and the dentate line determined before radiation is no longer accurate. An alternative way to determine the safe distal resection line after radiotherapy is to determine the safe distal resection margin length, that is, the distance between the visible tumor margin and the resection line. This length is based on the maximal tumor intramural spread distance in fresh tissue and can be determined by measuring the distance between the visible tumor margin under the naked eye and the microscopic tumor margin. However, in previous studies, the measurement of the intramural tumor spread distance was usually based on the distance between the microscopic tumor margin and the macroscopically visible tumor margin after formalin fixation [2, 3]. The tumor margin after fixation with formalin might not be the same as the visible tumor margin in vivo, which is still fresh before resection. This limits the direct application of intramural spread distance to determine a safe distal resection line in surgical procedures.

According to the literature, the safe distal resection margin length without preoperative radiotherapy has recently decreased from the original 5 cm [4] to shorter [5, 6], and even to 1 cm [7]. However, the intramural spread distance after radiotherapy may not be the same as that without radiotherapy; therefore, the resection margin length may also be different after radiotherapy. While, research about the intramural spread distance after radiotherapy is rare [2, 3].

Moreover, most studies have focused on the distal margin, and few have focused on the lateral margins. However, in the narrow pelvic cavity, the distal rectal transection line is often not perpendicular to the axis of the rectum to preserve more postoperative anorectal function [8], both the distal resection margins and the lateral ones are important for surgical oncological safety.

This study aimed to determine the safe post-radiation distal resection margin length during surgery, that is, the safe distance between the tumor visible margin during operation and the resection line. This length will be determined based on the maximal tumor intramural spread distance in fresh tissue, that is, the distance between the visible tumor margin under the naked eye before resection and the actual tumor margin only visible under the microscope after radiotherapy.

## Methods

This prospective study was performed at the First Affiliated Hospital of Naval Military Medical University from June 2018 to December 2018. The current research was approved by the Ethics Committee of the First Affiliated Hospital of Naval Military Medical University (committee’s reference number CHEC2022-021) and followed the precepts established by the Helsinki Declaration. All patients provided informed consent before surgery. We included a total cohort of 20 consecutive patients who fulfilled the following inclusion criteria: (1) older than 18 years, (2) diagnosed with middle or low rectal cancer, (3) had already undergone preoperative radiotherapy, and (4) intended to undergo radical resection. The exclusion criteria were as follows: (1) refusal to undergo surgery after radiotherapy; (2) clinical complete response and received “wait and watch” approach; (3) local resection after radiotherapy; and (4) patients who did not complete radiotherapy.

All patients received intensity-modulated long course radiotherapy in the case of preoperative stage T3 – T4 or N+, or if the circumferential margin was considered positive: a total dosage was 45 ~ 50.4 Gy (1.8 ~ 2.0 Gy per time, in 25 – 28 fractions). Oral capecitabine (825 mg/m^2^ twice/day) was prescribed concurrently with long-course radiotherapy in all patients according to the National Comprehensive Cancer Network (NCCN) guidelines. Radiotherapy was followed by 1 – 3 cycles of FOLFOX or CAPEOX before surgery. The preoperative treatment was performed at the Department of Medical Oncology. Subsequently, radical resection, including low anterior resection (LAR) or abdominal perineal resection (APR), was performed in patients at the Department of Colorectal Surgery 5 – 13 weeks after the last dose of radiotherapy, based on the evaluation of tumor response to preoperative radiotherapy.

### Determination of the tumor visible margin in vivo with naked eyes

The size of the tumor and the visible margin of the tumor were determined intraoperatively based on morphological abnormalities, which were based on palpation and the surgeons’ naked eyes. These morphological abnormalities can present as mucosal fold changes, poor mobility, and hardening texture, such as fibrotic scar tissue, characterized by red color, swelling, or ulceration.

### Labeling of the tumor visible margin with sutures and nanocarbon particles

**Fig. 1:**
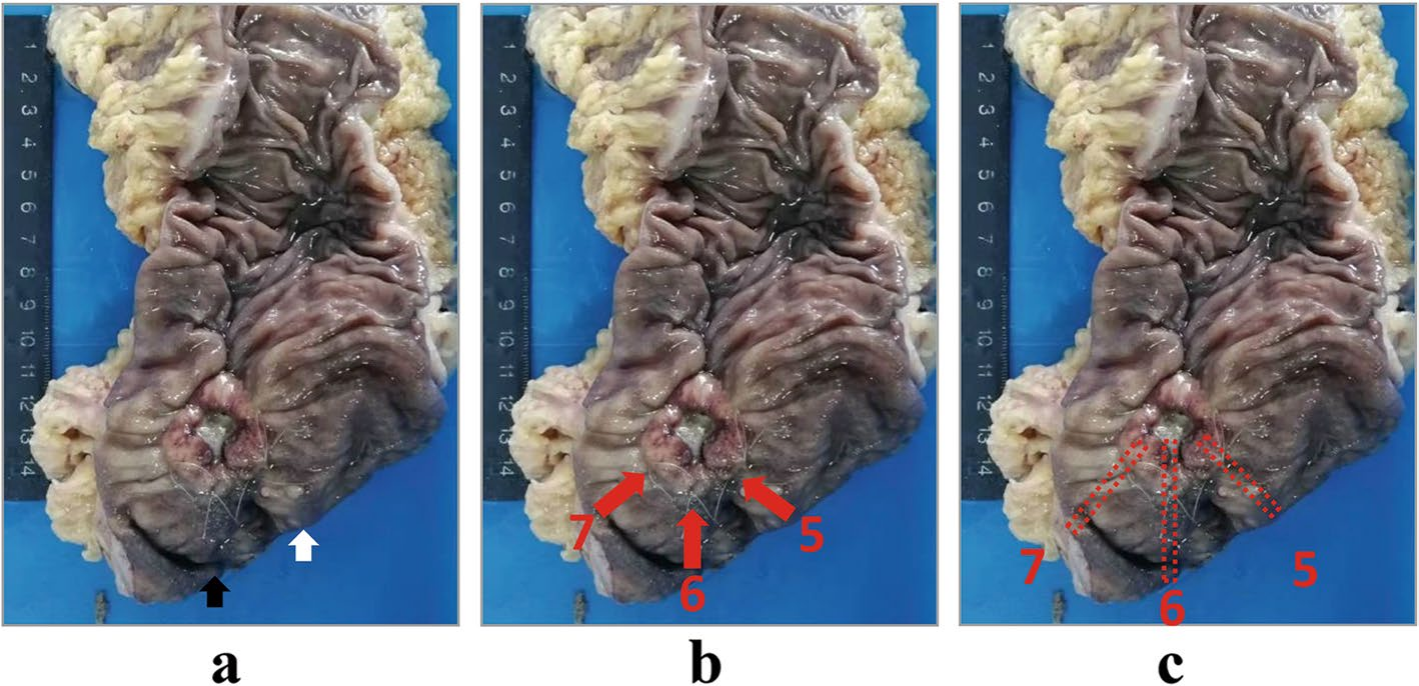
Labeling of the tumor visible margin with naked eyes. **A** Visible tumor margin was determined during operation and labeled with 5-0 sutures right after resection. The white arrow indicates the lateral resection margin, and the black arrow indicates the distal resection margin. **B** The red arrows marked with 5, 6, and 7 indicate the position labeled by sutures made at the tumor visible margins at 5, 6, and 7 o’clock. **C** The red squares marked with 5, 6, and 7 indicate the positions three tissues were retrieved from the specimen

Directly after resection, the intestine was routinely opened on the opposite side of the tumor. For the tumor involving 100% of the luminal circumference, we opened the intestine on the opposite side of the mesorectum. After reaching an agreement among the same group of colorectal surgeons regarding the position of the distal visible margin, the colorectal surgeons labeled the distal visible tumor margins at the distal 6, 5, and 7 o’clock positions using a 5 – 0 suture. The 6 o’clock position was the most distal tumor margin, this method is applicable as well for the tumor with 100% of the luminal circumference. The 5 and 7 o’clock positions were lateral tumor margins around 40 – 50° apart from the most distal margin on the right and left sides, respectively (Fig. 1). After being labeled with sutures, the specimen was fixed in 10% formalin for 24 h (Fig. 1a, b).

As indicated by the marking sutures, the fixed surgical specimens were injected with carbon nanoparticle suspension (Lummy, Chongqing, China) with volumes fewer than 0.02 ml, at the 5, 6, and 7 o’clock positions. A gauge 27 skin test syringe was used to reduce the influence of needle diameter on the results. We replaced the 5 – 0 sutures with carbon nanoparticles because the sutures would be lost during the subsequent cutting procedure of the specimen. The carbon nanoparticles were injected perpendicular to the surface of the specimen through the mucosa, submucosa, and muscle layers to mark the distal visible margin of the tumor according to the naked eye. The carbon nanoparticles facilitated the measurement of the tumor intramural spread distance under a microscope. The resection margin connected to a small amount of tumor tissue for comparison was collected in 2-cm-long blocks. Three tissue samples were collected from each patient along the nanocarbon markers at the 6, 5, and 7 o’clock positions (Fig. 1c). These three samples from each patient were used to investigate the intramural tumor spread distance under a microscope using the following procedure.

### Microscopic evaluation of the tissues

All samples were embedded in paraffin using a paraffin-embedding module (Leica EG1150, Leica, Germany) and cut into sections (4 μm thickness) in the longitudinal direction. Microscopic evaluation was performed using a light microscope (Leica DM4 B, Leica, Germany) connected to a digital camera (Leica DFC9000, Leica, Germany). Microscopic evaluation was performed only ex vivo, that is, on resected tissues. Hematoxylin and eosin staining was performed to evaluate the morphology of the tissue samples.

### Estimation of the distal intramural spread distance

**Fig. 2:**
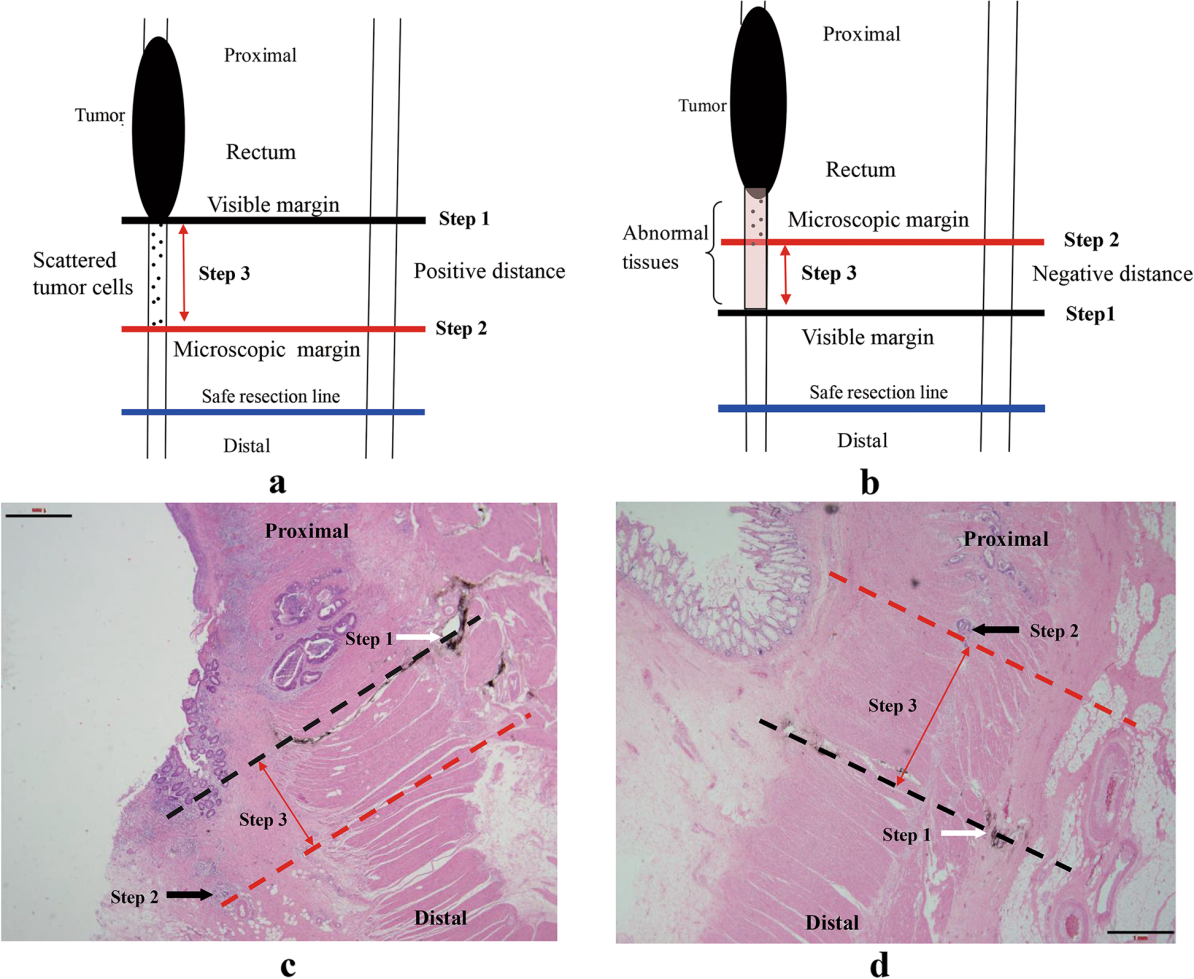
Estimation of the intramural spread distance between visible margin and microscopic margin. **A** Schematic explanation for positive spread distance, Step 1: the black line was visible tumor margin under naked eyes. Step 2: the red line was a microscopic tumor margin under the microscope. Step 3: red double arrow length was tumor cell intramural spread distance, which is the distance between the visible tumor margin and the microscopic margin. Black little dots between the visible margin and the microscopic margin were scattered tumor cells. The blue line was a safe distal resection margin. **B** Schematic representation of the negative spread distance. The red, black, and blue lines and small black dots indicate the same results as indicated in **A**. There was swollen abnormal tissue, so the visible tumor margin was labeled at the distal edge of the abnormal tissue. **C** The pathological explanation for positive spread distance, corresponding to **A**. White arrow was the carbon nanoparticles, which indicated visible tumor margins under naked eyes. Black arrow was the furthest scattered tumor cells. **D** The pathological explanation for negative spread distance is shown in **B**

The steps used to measure the distal-spread distance are shown in Fig. 2. The intramural spread distance was defined as the distance between the naked-eye visible margin and the microscopic tumor margin. Ex vivo intramural spread distance, i.e., distance between the carbon nanoparticles and distal tumor cells was measured ex vivo under the microscope for each slide. First, the carbon nanoparticles indicating the visible tumor margin under the naked eye were used as a zero point (Fig. 2a, black line as the visible margin, step 1). Second, based on the microscopic view, the distal tumor cells were found, and in this way, the distal tumor margin under the microscope (microscopic margin) was determined (Fig. 2a, red line as microscopic margin, step 2). Lastly, the distance between the naked-eye visible margin (black line) and microscopic margin (red line) was measured as ex vivo distal intramural spread distance (Fig. 2a, red double arrow length as ex vivo spread distance, step 3).

When the tumor cells were located proximal to the carbon nanoparticles, the distal spread distance was described as negative (Fig. 2b, d). If no tumor cells, but only mucus, were found, the measurement was not applicable and the value was recorded as as "NT". The distal intramural spread distance in vivo was the value ex vivo multiplied by 1.75 as a shrinkage factor according to the literature [9]. The tumor differentiation level, tumor deposits, tumor budding, perineural invasion, tumor regression grade, and T/N stage were also recorded according to AJCC 7th edition [10]. Tumor regression grade (TRG) was defined according to the scale proposed by Ryan et al., with 0 indicating no viable cancer cells, 1 indicating single cells/small group of cancer cells, and 2 indicating residual cancer outgrown by fibrosis, and 3 indicating extensive residual cancer [11]. Data acquisition was performed using the ImageScope v12.2.2.5015 software (Aperio, USA).

### Statistical analysis

All statistical analyses were performed using IBM SPSS Statistics, version 23.0 (Armonk, NY, USA). Continuous values were reported as mean ± standard deviation (SD) or median ± interquartile range (IQR), depending on whether the data were normally distributed. Categorical values were reported as absolute numbers and percentages. Continuous variables were compared using the *t* test for independent samples if the variables were normally distributed or the Mann-Whitney test if not normally distributed. Categorical variables were analyzed using the chi-squared test. Statistical significance was set at a two-sided *P* value < 0.05. Pearson’s test was used to analyze the correlation between the tumor intramural spread distance and the time interval between radiotherapy and surgery.

## Results

### Patient’s characteristics

Twenty consecutive patients were recruited for the study. The patients’ characteristics are presented in Table 1. Out of the 17 patients who received low anterior resection, 16 got loop ileostomy. The mean distance between the lower tumor margin to the anal verge was 4.8 ± 1.7 cm before radiotherapy. The mean time interval between radiation and operation was 65.9 ± 17.8 days. Of the 20 specimens in the current study, in 19 cases the tumor was an ulcer and in only one case it was an intra-luminal tumor, besides, 4 specimens involved 100% luminal circumference. All included patients were diagnosed with rectal cancers staged at ypT2 or ypT3, except one patient who was diagnosed with ypT1 stage cancer. Eleven patients (55%) were lymph node-negative, and 9 (45%) had positive lymph nodes. None of the patients showed a complete pathological response. All the patients underwent R0 resection.

**Table 1:** Patients’ characteristics (*n* = 20)

Variables	Value	Variables	Value
Age (years)	57.3 ± 14.4 ^a^	ypT stage	
Gender		T1	1 (5%)
Female	5 (25%)	T2	9 (45%)
Male	15 (75%)	T3	10 (50%)
Distance between distal tumor margin to anal verge before radiotherapy (cm)	4.8 ± 1.7 ^a^	ypN stage	
Time interval between radiation and operation (days)	65.9 ± 17.8 ^a^	N0	11 (55%)
Type of resection		N1/2	9 (45%)
APR	3 (15%)	M stage	
LAR	17 (90%)	M0	18 (90%)
Type of anastomosis		M1	2 (10%)
Stapler	16 (94.1%)	ypTNM Stage^c^	
Hand sewn	1 (5.9%)	I	7 (35%)
Stoma		II	5 (25%)
Diverting loop ileostomy	16 (80%)	III	6 (30%)
End colostomy	3 (15%)	IV	2 (10%)
No stoma	1 (5%)	TRG^d^	
cT stage		I	6 (30%)
T4	5 (25%)	II	11 (55%)
T3	13 (65%)	III	3 (15%)
T2	2 (10%)	Tumor involving 100% luminal circumference	
cN stage		Yes	4 (20%)
N0	4 (20%)	No	16 (80%)
N1/2	16 (80%)	Diameter of tumor (cm) ^e^	2.65 ± 1.05^a^
cTNM stage ^b^		Moderately differentiated	20 (100%)
I	1 (5%)	Tumor deposit	5 (25%)
II	3 (15%)	Tumor budding	4 (20%)
III	14 (70%)	Perineural invasion	4 (20%)
IV	2 (10%)	No. of retrieved lymph nodes	9.9 ± 1.0^a^

^a^Mean ± SD

^b^cTNM stage: clinical stage before radiotherapy according to AJCC 7th edition [10]

^c^ypTNM Stage: pathological stage after radiotherapy according to AJCC 7th edition [10]

^d^TRG: tumor regression grade according to Ryan et al. [11]

^e^The longest diameter of the tumor

### Incidence of distal intramural spread > 0 mm

Overall, ten patients (50%) had distal intramural spread > 0 mm. Three patients had distal intramural spread at one point, six patients at two points, and one patient at three points. We found that 7(35%), 5(25%), and 6(30%) patients had distal tumor spread at the 5, 6, and 7 o’clock positions, respectively. Detailed information about the distal intramural spread is shown in Table 2.

**Table 2:** Position and frequency of distal intramural spread (*n* = 10)

Distal intramural spread position^a^	Number of patients	Proportion of all the patients (%)
5 clock	1	5%
6 clock	1	5%
7 clock	1	5%
5 and 6 clock	2	10%
5 and 7 clock	3	15%
6 and 7 clock	1	5%
5, 6 and 7 clock	1	5%

^a^The distal tumor cell intramural spread distance > 0 mm

### Layer and mode of the tumor intramural spread

For the ten cases with intramural spread, 50% (5/10) of the farthest spread was in the submucosal layer, and 50% (5/10) was in the muscle layer. Ninety percent (9/10) of cases were in the form of direct spread. One (10%) was in the form of microscopic foci that were discontinuous from the primary lesion. Of the 4 patients with maximal *ex vivo* tumor spread distance of 0.5 cm, 3 (75%) had tumor budding, perineural invasion, tumor deposit, or lymph vascular invasion (Table 3).

**Table 3:** Mode and layer of the distal intramural spread > 0 mm (*n* = 10)

Patient	Tumor budding	Perineural invasion	Tumor deposit	lymph vascular invasion	TNM	Tumor differential	Layer of spread^a^	Mode of spread^a^	Location of longest spread (o’ clock)	Longest distance (cm)^b^
1	−	−	−	−	T2N0M0	Moderate	Submucosa	Direct spread	5	0.1
2	−	+	−	−	T3N0M0	Moderate	Submucosa	Direct spread	5	0.5
3	+	−	+	−	T3N2aM1	Moderate	Submucosa	Direct spread	7	0.4
4	−	−	−	−	T3N0M0	Moderate	Muscle layer	Direct spread	7	0.4
5	+	−	+	−	T2N1aM0	Moderate	Submucosa	Direct spread	5	0.5
6	+	+	+	+	T3N2bM1	Moderate	Muscle layer	Direct spread	6	0.2
7	−	+	−	−	T3N0M0	Moderate	Muscle layer	Foci discontinuous	6	0.4
8	−	−	−	+	T2N1aM0	Moderate	Muscle layer	Direct spread	5	0.5
9	−	−	−	−	T2N0M0	Moderate	Submucosa	Direct spread	5	0.5
10	−	+	−	+	T3N2aM1	Moderate	Muscle layer	Direct spread	6	0.2

^a^If the intramural spread occurred at more than one sites, then the largest value is used

^b^The tumor cell intramural spread distance ex vivo

### Estimation of the tumor spread distance

The intramural distal-spread distances measured ex vivo for each patient are reported in Table 4. The intramural tumor spread distance in the only case with an intraluminal tumor was − 0.1, NT, and − 0.2 cm at 6, 5, and 7 o’clock, respectively, under the microscope.

**Table 4:** The distal spread distance of tumor cell under the microscope for individual patients (*n* = 20)

Patients	5 o’clock (cm)	6 o’clock (cm)	7 o’clock (cm)
1	< − 0.4*	< − 0.4*	< − 0.3*
2	− 0.2*	0	− 0.3*
3	− 0.6*	0	0
4	0.1	− 0.2*	− 0.4*
5	0.5	− 0.4*	0.3
6	< − 0.8*	NT**	− 0.6*
7	NT**	NT**	NT**
8	− 0.1*	NT**	0.4
9	0.1	− 0.2*	0.4
10	0.5	0	0.4
11	− 0.3*	− 0.3*	− 0.2*
12	0	− 0.5*	NT**
13	NT**	NT**	NT**
14	NT**	− 0.1*	− 0.2*
15	NT**	0	− 0.2*
16	0.1	0.2	0
17	− 0.1*	0.4	0.1
18	0.5	0.1	> 0.3
19	0.5	0.4	NT**
20	− 0.1*	0.2	NT**

*Minus (−) means that the spread of tumor cells did not cross the position of the nanocarbon particles marks distally

**NT means that no tumor cells were left, only some mucus, ulcer, or epithelial dysplasia were seen

At the 6 o’clock position, the longest distal spread distance ex vivo was 4 mm, thus the longest distal spread distance in vivo was 4 × 1.75 (shrinkage factor) = 7 mm. The mean spread distance in vivo at 6 o’clock was − 0.9 ± 4.8 mm with 95%CI for the mean being − 3.4 ~ 1.7 mm. At 5 o’clock, the longest distal spread distance in vivo was 8.8 mm, and the mean value in vivo was − 0.3 ± 6.9 mm with 95%CI for the mean being − 4.0 ~ 3.4 mm. Finally, at 7 o’clock, the longest spread distance in vivo was 8 mm and the mean value in vivo was − 0.4 ± 5.7 mm with 95%CI for the mean being − 3.5 ~ 2.8 mm.

### The correlation between the tumor spread distance and the time interval from the completion of radiotherapy to operation

**Fig. 3:**
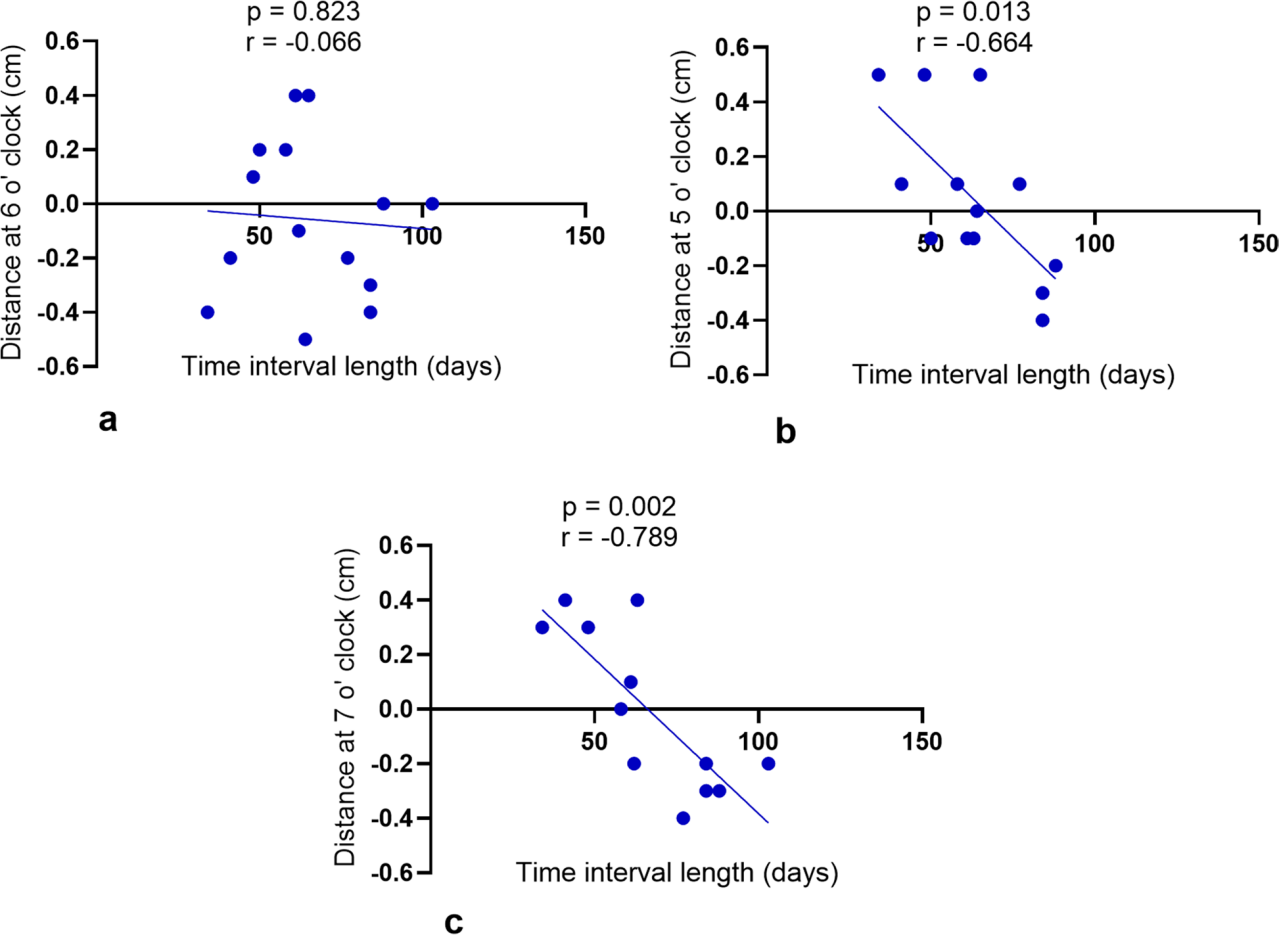
The correlation of the interval length from the completion of the radiotherapy to operation and the tumor intramural spread distance at **A** 6 o’clock; **B** 5 o’clock; and **C** 7 o’clock

The time interval from the completion of radiotherapy to operation was negatively correlated with the tumor spread distance at 5 o’clock (*r* = − 0.664, *p* = 0.013) and 7 o’clock (*r* = − 0.789, *p* = 0.002), but not correlated with the distance at 6 o’ clock (Fig. 3).

## Discussion

The present study showed that the maximal tumor intramural spread distance after radiotherapy under the microscope at 5, 6, and 7 o’clock positions was 5, 4, and 4 mm, respectively. According to the theory of Goldstein NS [9], the intestine length ex vivo determined pathologically should be multiplied by a shrinkage factor of 1.75 for interpreting the intestine length in vivo; thus, the maximal tumor intramural spread distance in vivo during operation at the 5, 6, and 7 o’clock positions were 8.75, 7, and 7 mm, respectively.

A prospective study by Weese et al. [12] showed that the method of measuring distal margin length requires a specific definition, as different techniques provide different results. Sondenna et al. compared distal resection margin length using different methods of measurement [13] and measured the distal margin prospectively in five different ways in 20 patients. Their results showed different results according to different methods. In this study, we investigated the maximal *ex vivo* spread distance after formalin fixation using a microscope. Then, we multiplied the ex vivo spread distance with a shrinkage factor of 1.75 to get the maximal spread distance in vivo. To measure the intramural spread distance accurately in the current study, first, labeling straight after the resection in “fresh” tissue by the surgeon is more accurate and provides direct guidance for clinical practice. To increase the reliability of the labeling of the visible margin, two colorectal surgeons should reach an agreement. Second, the carbon nanoparticles should be injected at the suture points after fixation of the specimen instead of before fixation, because the carbon nanoparticles in fresh tissues will diffuse into the lymphatic ducts, which will make the measurement of the distance between the carbon nanoparticles and the tumor cells under the microscope impossible and inaccurate. Third, the distal intramural spread distance ex vivo under the microscope was multiplied by 1.75 to get the value in vivo [9].

Distal spread of tumor cells might exist in a layer without involving the mucosa, which was the only part that was directly visible to the surgeon’s eyes. Thus, it is important to know the possible intramural spread distance in advance, especially after radiotherapy. Much research has been carried out on distal intramural spread [14]. With the development of neoadjuvant chemoradiotherapy and the corresponding interest in sphincter preservation, the intramural spread distance should be re-evaluated to preserve more sphincters and achieve oncological safety for patients with low rectal cancer. However, distal intramural spread after preoperative chemoradiotherapy for rectal cancer has not been studied extensively [15].

For the length of distal intramural spread without preoperative radiotherapy, Madsen et al. found in 1986 that the extent of distal intramural spread is generally less than 10 mm; only 25% of 43 rectal cancer patients had over 5 mm intramural spread in pathological observation, and 18.6% had over 10 mm intramural spread [14]. For the intramural spread distance after preoperative chemoradiotherapy, Guillem et al. assessed 109 patients with mid to low rectal cancer, and distal intramural spread of cancer was shown to be within 0.95 cm in pathological observation [2]. Guedj et al. analyzed 124 specimens after preoperative radiotherapy, and only one patient had a distal spread of over one centimeter [3]. However, these distal-spread distances were all measured based on the visible tumor margin after fixation with formalin, which is different from the judgment of the tumor visible margin in fresh tissues. In our analysis, we found that the farthest distal spread distances after radiotherapy in the distal and lateral directions in vivo were 7 and 8.75 mm. This length is consistent with the previous literature [3].

According to the literature, the extension of distal intramural spread beyond 10 mm is associated with numerous lymph node metastases and advanced tumors [14, 16, 17]. This is consistent with our study that out of the 4 patients with an intramural spread distance of 8.75 mm in vivo, 3 patients had tumor budding, perineural invasion, tumor deposit, or lymph vascular invasion. Williams et al. suggested that distal tumor intramural spread should be regarded as a more systemic spread rather than a regional one, which means that increasing the distal resection margin length cannot prevent distant metastasis when the distal intramural spread distance is > 0 mm [18]. Vernava et al. proposed that local recurrence was not significantly different when the distal resection margin length was < or > 1 cm. However, patients with distal resection margin length < 0.8 cm, had more local recurrence [19]. The longest intramural spread distance in the current study was 0.875 cm, which might explain why a distal resection margin < 0.8 cm indicated higher local recurrence in the study by Vernava et al. [19]. In the current study, a distal resection margin (0.7 cm in vivo at 6 o’clock after radiotherapy was sufficient from an oncological point of view. Besides, we hypothesize that when running to the patients with distal intramural spread distance > 0.7 cm, even if the resecting distal margin was longer than 0.7 cm, it may still be of no help in improving the oncological outcome, especially in preventing distant metastasis. Considering the maximal intramural spread distance of 8.75 mm in vivo at 5 o’clock in the current study, a rounder value of no less than 1 cm is sufficient for resection margin length without deteriorating overall survival.

We found that the time interval between radiotherapy and surgery was not correlated with the intramural tumor spread distance at the 6 o’clock position but correlated with the intramural spread distance at the 5 and 7 o’clock positions. This might indicate further tumor shrinkage after a longer interval between radiotherapy and surgery. In addition, more rectal walls and anal canal might be preserved in the lateral rectum wall so that better anal function could be preserved after the operation [8]. However, the current study has a small sample size, we cannot draw a firm conclusion on the influence of the time interval length after radiotherapy on the distal tumor intramural spread distance.

There were also some limitations to the current study; the number of patients was relatively small. Additionally, this study was conducted at a single institution, incorporating the risk of selection bias. In addition, the outcome of shorter distal resection margins in patients undergoing preoperative chemoradiotherapy requires further follow-up and comparison studies.

## Conclusions

The intraoperative distance between the distal resection line and the visible margin of the rectal tumor after radiotherapy should not be less than 1 cm to ensure oncological safety. Further studies with larger sample sizes and follow-up periods should be conducted to validate this value.

## Data Availability

The datasets for the current study are available from the corresponding author upon reasonable request.
